# Solitary Fibrous Tumor in the Perianal Region: Report of Two Cases With an Atypical Location

**DOI:** 10.7759/cureus.12887

**Published:** 2021-01-24

**Authors:** Jorge Alejandro Gonzalez, Elio Sanchez, Oscar Messa Botero, Sergio Cervera-Bonilla

**Affiliations:** 1 General Surgery, Pontificia Universidad Javeriana, Bogota, COL; 2 Surgical Oncology, Instituto Nacional de Cancerología, Bogota, COL; 3 Oncologic Pathology, Hospital Universitario San Ignacio, Bogota, COL; 4 Breast and Soft Tissue Surgery, Hospital Universitario San Ignacio, Bogota, COL; 5 Breast and Soft Tissue Surgery, Instituto Nacional de Cancerología, Bogota, COL

**Keywords:** solitary fibrous tumor, perianal, perineal

## Abstract

A solitary fibrous tumor (SFT) is a mesenchymal neoplasm of spindle cells, initially described in the pleura. The World Health Organization (WHO) classifies the solitary fibrous tumor as a neoplasm with intermediate biological potential. Diagnostic images are essential for the diagnostic and therapeutic approach in this entity. The standard of treatment for this type of lesion is surgical resection with oncological margins larger than 1 cm. The solitary fibrous tumors located in the perianal, perineal, and pelvic regions are infrequent and represent a challenge in the clinical approach, mainly because the manifestations are nonspecific. Given the low incidence of this type of neoplasm, we present two cases of SFT in the perianal region managed in a high-complexity hospital.

## Introduction

A solitary fibrous tumor (SFT) is a mesenchymal neoplasm of spindle cells, which was initially described in the pleura and has received different names according to the histological findings that are observed; however, it has also been described in other locations such as the abdominal region and soft tissues [1-3]. The World Health Organization (WHO) classifies the SFT as a neoplasm with intermediate biological potential (relatively indolent with a low risk of metastasis), and it usually has benign behavior [1-2,4]. Several studies have attempted to identify clinical and paraclinical factors that allow the differentiation of the SFTs with benign behavior from those that behave more aggressively and with malignant characteristics; among them stand out: age, location, large size tumors, high mitotic rate (>4/10 HPF), high cellularity, and cell pleomorphism [2,5-6]; however, there is much controversy and heterogeneity in the available literature regarding the factors associated with malignancy and increased risk of relapse. Moreover, it has been observed that tumors of benign behavior can relapse locally.

The scarce knowledge of tumor biology and the low incidence of this type of tumor has generated great uncertainty about medical and surgical treatment strategies. It has been described that surgical resection remains the standard treatment, however, approximately 20% of the patients who are taken to surgical treatment have a subsequent local recurrence [7]; for this reason, the use of adjuvant therapy in SFT has been proposed from some time ago. This knowledge gap was studied by the Rare Cancer Network in 2014 when the study on the role of adjuvant therapy in scarcely studied neoplasms such as SFT was started.

Given the low incidence of this type of neoplasm, we present two cases of SFT in the perianal region managed in a high-complexity hospital.

## Case presentation

Case 1

**Figure 1:**
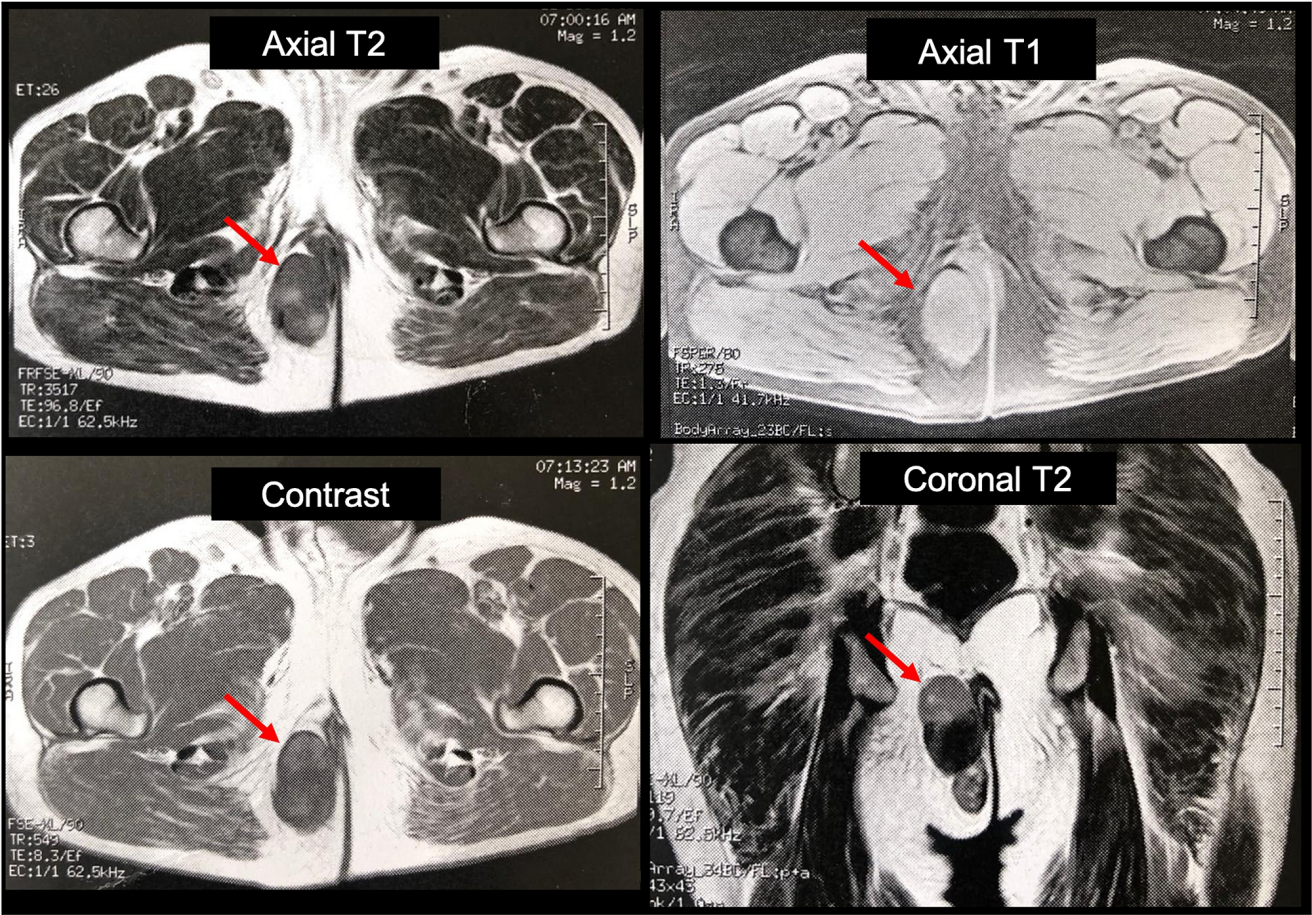
Contrasted MRI Mass with lobulated contours in the right ischioanal base whose dimensions were 36 x 65 x 74 mm, in close contact with the external anal sphincter without contact with the skin of the gluteal fold (red arrow) MRI: magnetic resonance imaging

A 57-year-old male patient who consulted for clinical symptoms of six months of evolution, with a mass in the right gluteal region that prevented him from sitting, with a progressive increase in size, associated with pain, limited walking, and a slight difficulty in defecation. On physical examination, a mass of 8 x 7 cm was found in the lower inner quadrant of the right gluteus, in close contact with the perianal region, mobile, not attached to deep planes, and with no evidence of regional adenopathies. A mass with partially defined, irregular contours of heterogeneous echogenicity, predominantly hypoechoic, located in the medial region of the right gluteus in the subcutaneous cellular tissue, measuring 64 x 45 mm was observed in the ultrasonography; the MRI of the pelvis with contrast (Figure 1) revealed a mass with lobulated contours in the right ischioanal base and the subcutaneous cellular tissue of the homolateral gluteal fold, whose dimensions were 36 x 65 x 74 mm, in close contact with the external anal sphincter, slightly displaced medially without contact with the skin of the gluteal fold.

With these findings, desmoid fibromatosis and soft tissue sarcoma were proposed as the differential diagnoses. A TRU-CUT biopsy was performed, whose pathology report described a lesion compatible with a solitary fibrous tumor presenting a proliferation of spindle cells and small cells, positivity for CD34, CD99, BCL2, and STAT-6 with less than one mitosis x 10 high-power field (HPF), without necrosis and with Ki-67 lower than 10% and negativity for CD31, b-catenin, CK, desmin, CD117, and S100.

**Figure 2:**
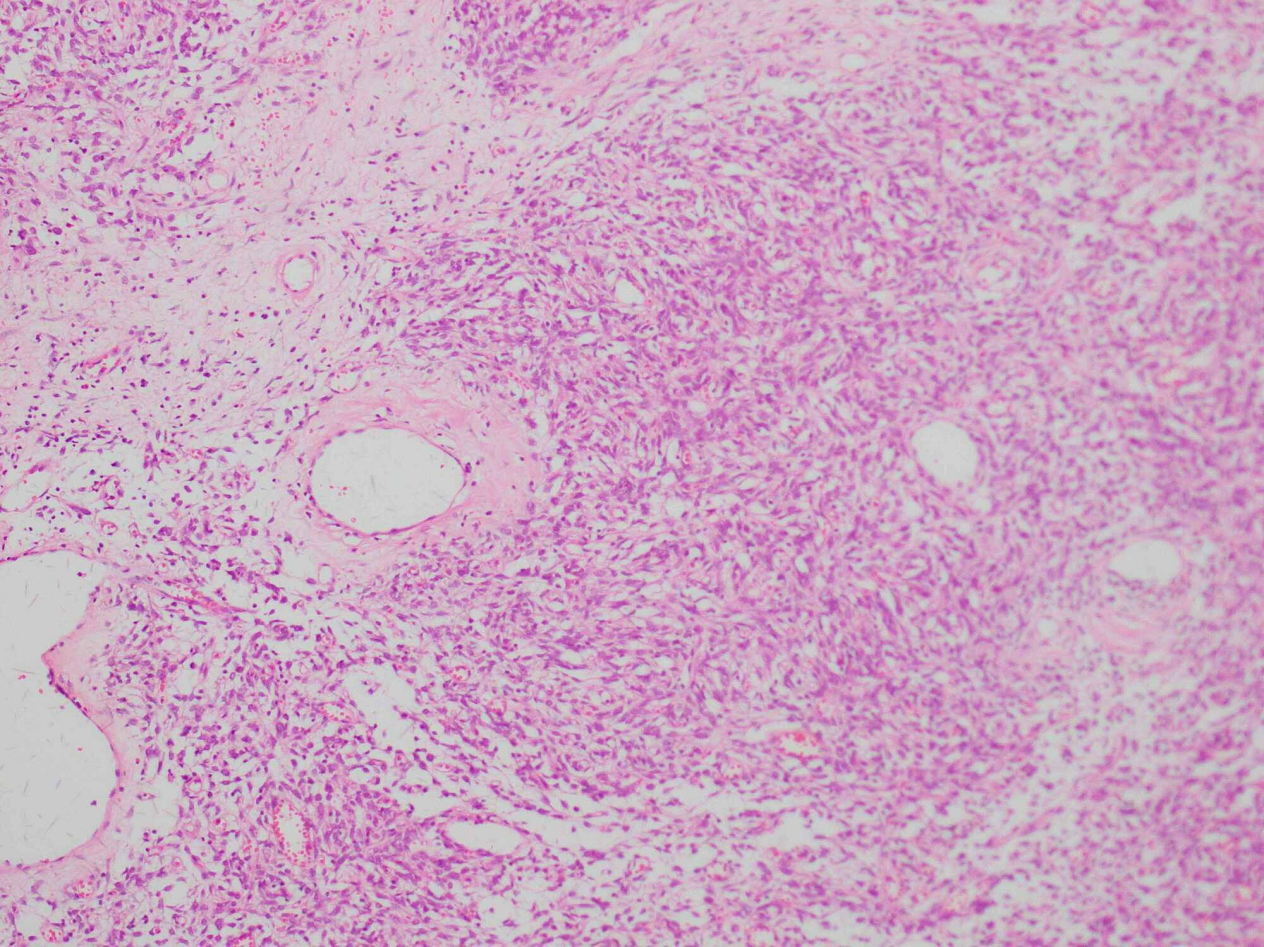
Hematoxylin & eosin, 10x Hypo and hypercellular areas without a specific growth pattern

**Figure 3:**
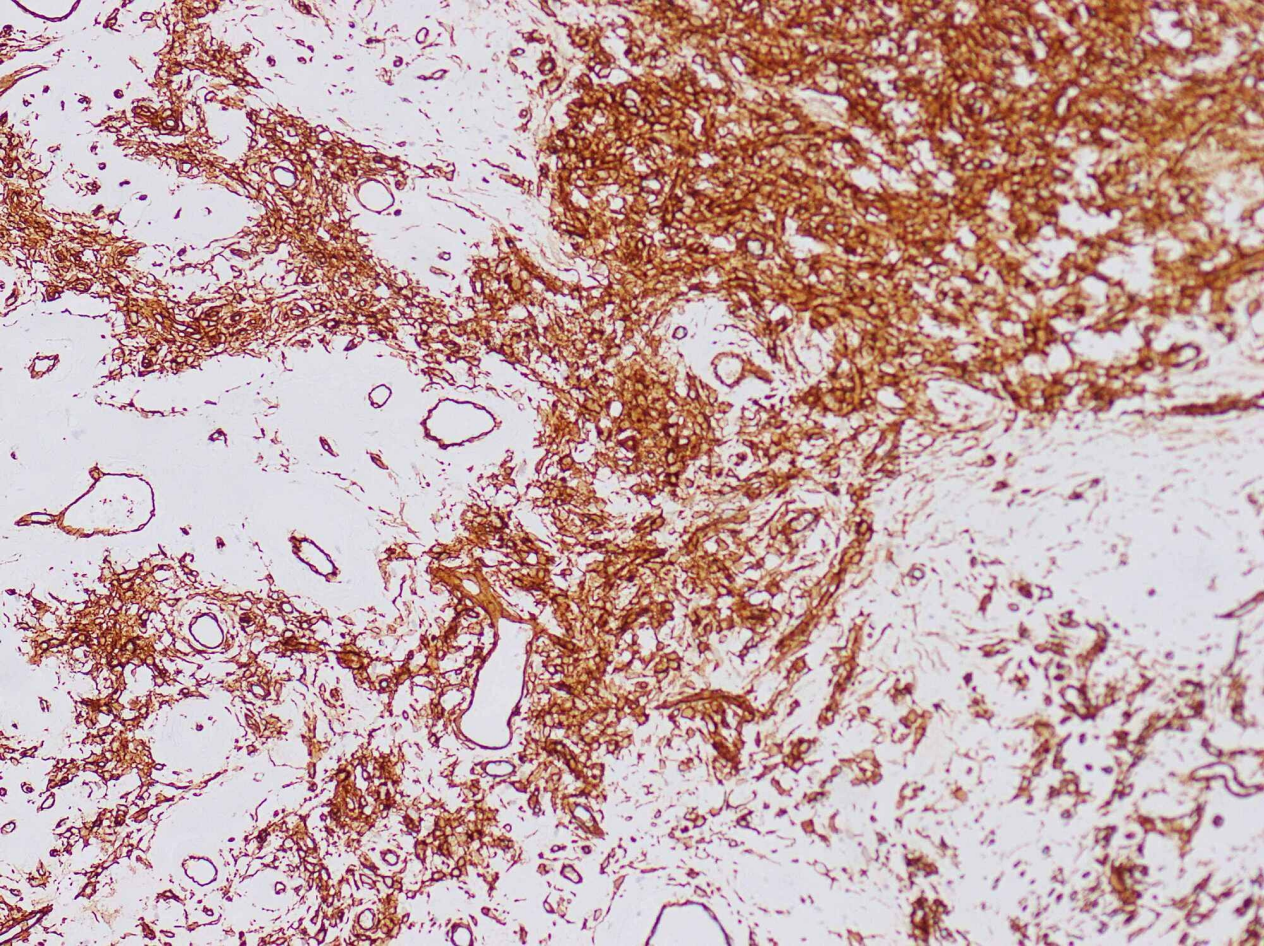
CD34, 10x Positive diffuse marking for CD34

Given that the clinical and histological findings showed good prognostic factors such as a tumor size of less than 10 cm, low mitosis rate, and age, it was decided to take him to surgical resection with the open perineal approach, with oncological margins. Within the intraoperative findings, a hypervascular fibrotic mass was identified in the right ischioanal region in close contact with the anal sphincter that required partial resection of the sphincter with immediate reconstruction. The anatomopathological study described a spindle cell lesion measuring 7.9 x 4.5 cm with hypo and hypercellular areas without a specific growth pattern, with prominent branched vessels with peripheral sclerosis (Figure 2). The section edge was at 1 mm from the ink. The immunohistochemical study showed reactivity for BCL2, CD34, CD99, and STAT-6 (Figure 3) with negativity for CD31, desmin, and cytokeratin AE1AE3. We found up to 15 mitoses x 2 HPF, with no evidence of necrosis, and no dedifferentiation was identified. With these findings, a solitary fibrous tumor of intermediate-risk was considered.

In the one-year follow-up with magnetic resonance imaging (MRI) of the pelvis and computed tomography (CT) scans of the thorax and abdomen, no clinical or imaging signs of locoregional or distant relapse have been identified and there was no compromise of fecal continence.

Case 2

A 50-year-old female patient, with no relevant medical antecedents, consulted for clinical symptoms of approximately one year of evolution consisting of anal pruritus, rectorrhagia, and perianal pain. On physical examination, a mass of 4 x 4 cm was found in the right ischiorectal fossa, mobile and painful on palpation, with no alterations in the assessment of the anal sphincter.

**Figure 4:**
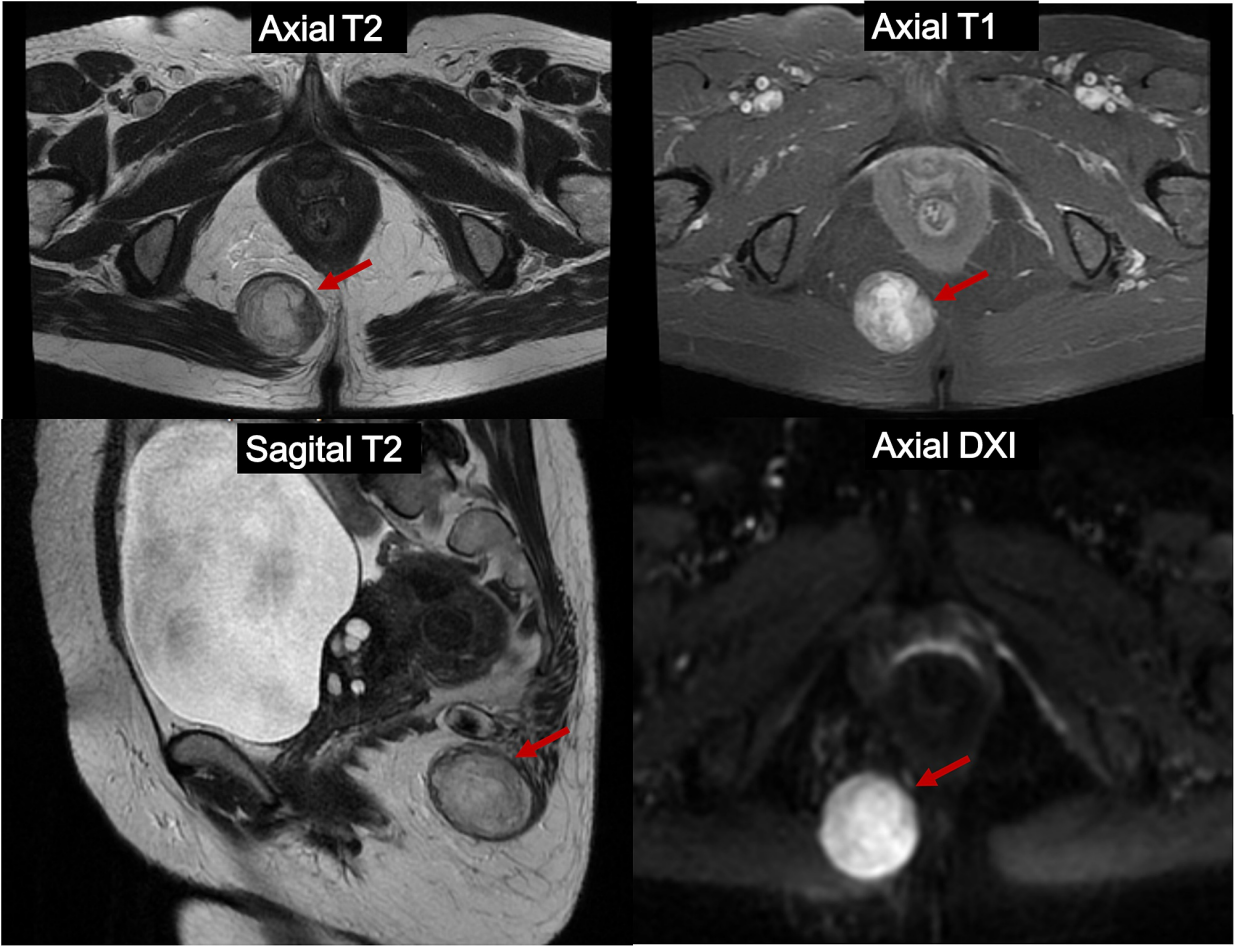
Contrasted MRI Well-defined oval lesion measuring 24 x 35 x 37 mm in the right ischiorectal fossa (Red arrow)

Regarding the diagnostic images, in the MRI of the pelvis (Figure 4), a well-defined oval lesion measuring 24 x 35 x 37 mm was evidenced in the right ischiorectal fossa and in contact with the gluteus maximus muscle, without the involvement of the rectum. In the CT scans of the thorax and abdomen, there was no evidence of lesions, suggesting distant disease. The rectosigmoidoscopy showed the presence of an extrinsic compression 50 mm from the anal margin.

The TRU-CUT biopsy was performed, whose result described the presence of a myxoid neoplasm of small cells, with an immunohistochemistry report of positivity for STAT6 and CD34 and negativity for desmin, CD10, CD56, calponin, S100, AE1/AE3, CK7, CK20, and P63. There was no evidence of mitosis or necrosis.

**Figure 5:**
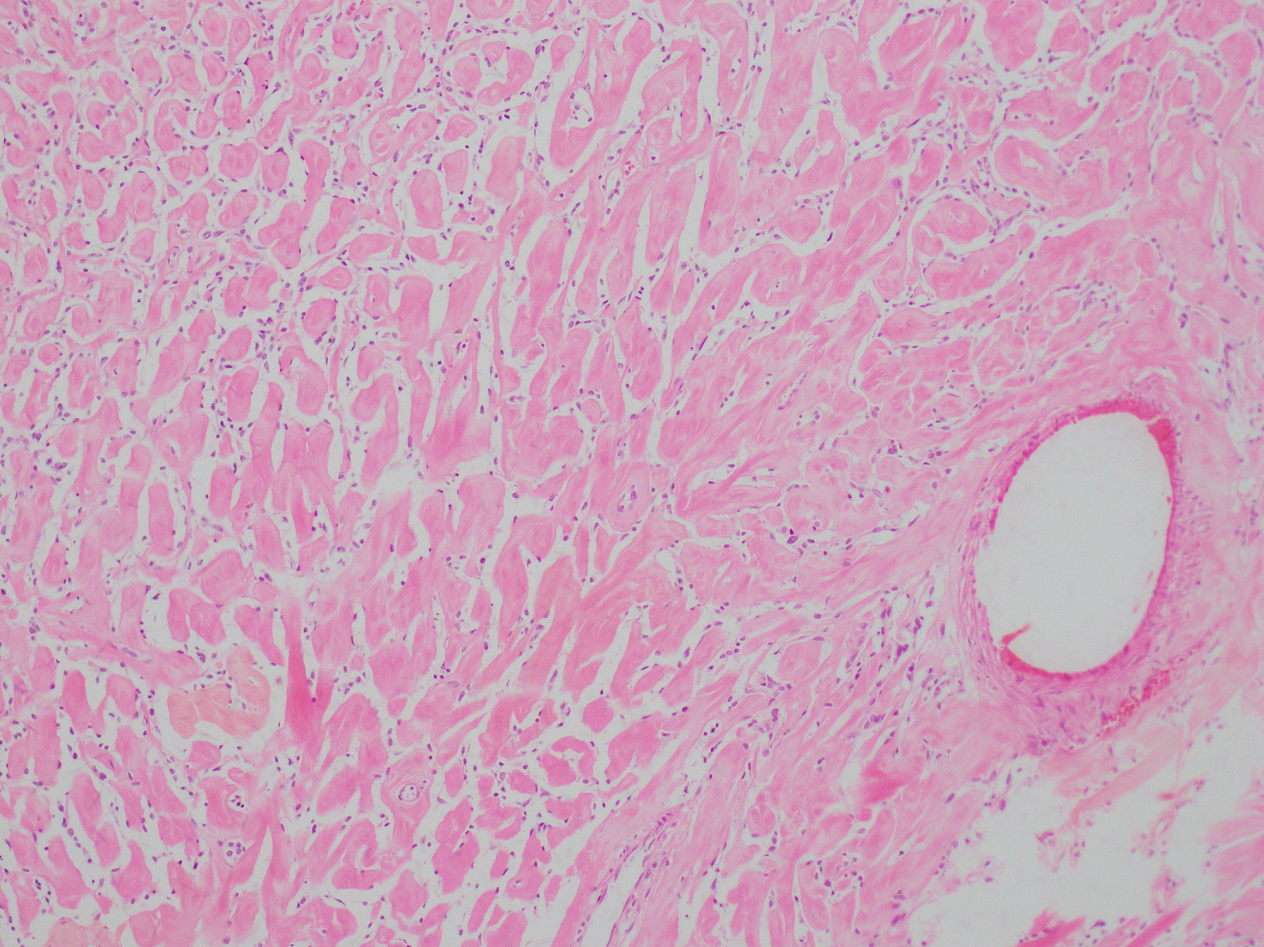
Hematoxylin & eosin, 10x Spindle-cell type, predominantly hypocellular with abundant extracellular collagen

**Figure 6:**
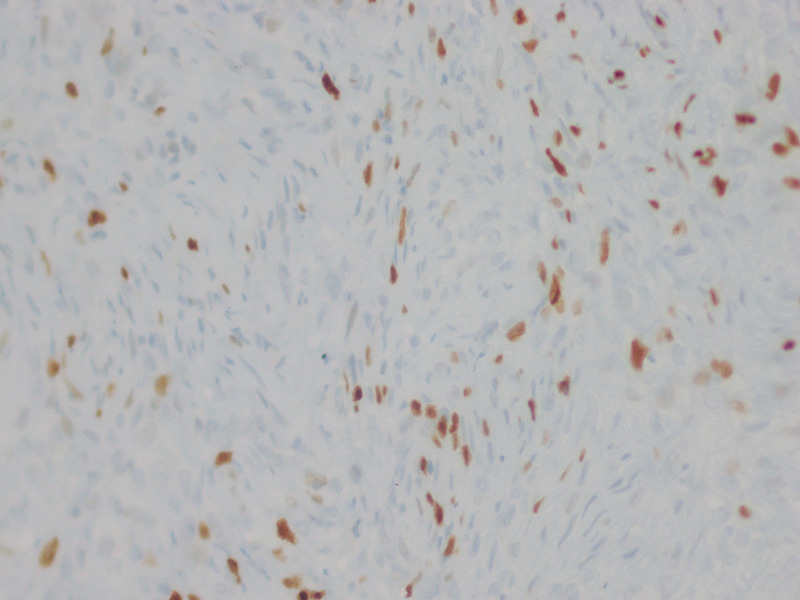
STAT6, 40x Intense nuclear reactivity was found for STAT6

The patient was taken to surgical resection by perineal route with oncological margins of 1 cm, with findings of a hypervascular mass measuring 4 cm, without the commitment of the rectal wall or the sphincter complex. The anatomopathological findings described a tumor of 4 x 3 cm, of spindle-cell type, predominantly hypocellular with abundant extracellular collagen (Figure 5) with less than four mitoses x 10 HPF, without the presence of necrosis or anaplasia, and no dedifferentiation areas were identified; the immunohistochemical study showed reactivity for CD34 and STAT-6 (Figure 6) with negativity for S-100, SOX-10, and CD31. With these findings, it was concluded that it was a solitary fibrous tumor of low risk.

In the two-year follow-up with MRI of the pelvis and thorax and abdomen CT-scans. No clinical or imaging signs of locoregional or distant relapse have been identified and there was no compromise of fecal continence.

## Discussion

The first report describing the localized nature of the SFT dates from 1870 and was made by Wagner in a tumor of pleural origin; however, it was not recognized until the year 1931 with the publications of Klemperer and Rabin [8]. Since then, it has received multiple names, such as a localized fibrous tumor, localized fibrous mesothelioma, localized pleural mesothelioma, fibrous mesothelioma, localized fibrous mesothelioma, pleural fibroma, subserous fibroma, and sub-mesothelioma fibroma [4].

The most used term was hemangiopericytoma (HPC), described by Murray in 1942 [1]. It was used to describe tumors with certain histological characteristics such as monotonous appearance, moderate cellularity and branched blood vessels with thick walls, and morphology of “reindeer horns.” These findings were described in pleural lesions, but by the year 1949, it was demonstrated that this type of tumor can be found anywhere in the soft tissues [2].

The age of presentation ranges between 20 and 80 years, its diagnosis in the fifth and sixth decades of life being more frequent. The main locations of these lesions are in the pleura and at the abdominal level (60%-70%). They have also been described in deep soft tissues but without identifying differences in terms of outcomes [6]. Clinically, they manifest as painless slow-growing masses, with symptoms that vary according to their location by compromise or displacement of other structures [9]. They are genetically associated with the fusion of the genes NAB2-STAT6 in chromosome 12q13. In the context of SFTs, the NAB2 fusion inherits a domain of activation of the signaling molecule STAT6, which converts a transcriptional repressor (NAB) into a potent transcriptional activator (i.e., NAB2-STAT6) of the EGR1 (early growth response protein), causing activation of EGR-mediated transcription, which culminates in a feedback process that causes cell growth with the secondary neoplastic progression [10].

Diagnostic images are essential for the diagnostic and therapeutic approach in this entity. In the cases presented, a study with magnetic resonance imaging of the pelvis was conducted in order to establish the size, behavior, characteristics, locoregional commitment, and differential diagnoses. The most frequent imaging findings are the presence of well-defined masses that tend to displace adjacent structures with smooth margins, very well vascularized, and that may have central necrosis or hemorrhage, very rarely calcifications, and in a low percentage (<9%) with local involvement. The presence of fatty tissue is suggestive of lipomatous hemangiopericytoma [11-12].

The World Health Organization (WHO) categorizes this neoplasm as with intermediate biological potential (low metastatic risk) [5-6]. Tumor size, mitotic rate, cellularity, pleomorphism, and age have been described as predictors of malignancy. Patients with tumors larger than 10 cm, more than four mitoses in 10 high-power fields, and over 55 years of age require a stricter follow-up due to the high risk of metastasis and death [6-7].

The standard of treatment for this type of lesion is surgical resection with oncological margins larger than 1 cm. However, there is no standardized treatment for this type of lesion given the scarcity of randomized studies, so treatment for sarcomas is taken as a reference. A study conducted by Demicco et al. [5] described a series of 110 patients with SFT, finding the presence of locoregional relapse in 10% of the patients, relapse with metastases in 26%, mainly in the lung and the liver, with disease-free survival at five to 10 years of 89 and 73%, respectively, and a distant disease-free survival at five to 10 years of 74 and 55%, respectively [6].

Recent studies have demonstrated good results in locoregional control in combination with surgery and radiotherapy. Bishop, in a retrospective study of patients with SFT who received surgery and perioperative radiotherapy, found excellent outcomes, with a five and 10-year survival of 95% and 89%, respectively, with a single tumor-related death due to lung metastasis, without evidence of local relapse [13]. In 100% of the patients analyzed, no locoregional relapse occurred at five and 10 years, however, it is a retrospective study, which limits it. In another retrospective study published in July 2020, preoperative and postoperative radiotherapy were compared, with no evidence of significant differences. However, in the local control, benefits of perioperative radiotherapy were observed, without affecting overall survival, mainly in those with less favorable resections and greater mitotic activity [14].

The SFTs located in the perianal, perineal, and pelvic regions are infrequent [12,15-16], and represent a challenge in the clinical approach, mainly because the manifestations are nonspecific. It has also been described that in up to 5% of the patients with SFT, refractory hypoglycemia can appear as a paraneoplastic syndrome (Doege-Potter syndrome). It is relevant to take into account among the differential diagnoses other types of tumors, primary anorectal tumors, prostate tumors, and of sacral origin, in addition to other types of mesenchymal tumors such as aggressive angiomyxoma, neurofibromas, gastrointestinal stromal tumor (GIST), leiomyomas, liposarcomas, as well as metastatic tumors or lymphomas [16].

The surgical approach varies according to the size and location, being used most frequently in the posterior approaches, in the jackknife position, or the modified Kraske´s approaches to access the presacral space, ischiorectal, or pre-rectal fossae. In case of suspicion of hypervascularization based on the imaging findings, preoperative tumor embolization may be chosen in order to reduce the size and avoid the possible greater blood loss during resection.

## Conclusions

A solitary fibrous tumor is an extremely rare sarcoma. We presented two cases of atypical locations of this tumor, which were treated with oncological surgical resection, with no evidence of locoregional relapse in subsequent controls. The evidence demonstrates that surgical resection plus radiotherapy has good outcomes regarding the locoregional relapse, especially in those tumors with risk factors, which are not present in the presented patients. We consider that the treatment of choice for these patients was adequate, with excellent results to date and without the need for perioperative radiotherapy and its possible complications.
